# Supplementary material for: Formation of Natural Magnesium Silica Hydrate (M-S-H) and Magnesium Alumina Silica Hydrate (M-A-S-H) Cement
Source: Materials (Basel). 2024 Feb 21;17(5):994. doi: 10.3390/ma17050994 (PMC10934351; doi:10.3390/ma17050994)
Supplement: Supplementary file 1 [file materials-17-00994-s001.zip › materials-2812603-supplementary.pdf]

Table S1. Composition of brucite from Feragen.

Sample	Fer1-12	Fer1-12	Fer7-12
SiO2	2,81	0,83	0,16
TiO2	0,00	0,00	0,00
Al2O3	0,01	0,00	0,01
FeO	15,00	6,28	9,75
MnO	0,62	0,29	0,30
MgO	55,19	61,98	60,10
CaO	0,00	0,07	0,07
Na2O	0,00	0,00	0,00
K2O	0,00	0,00	0,00
Cr2O3	0,01	0,06	0,00
NiO	0,54	0,06	0,60
Total	74,16	69,56	70,95
Structural formulae based on 2(O)			
Si	0,0554	0,0167	0,0033
Ti	0,0000	0,0001	0,0000
Al	0,0002	0,0000	0,0001
Fe	0,1236	0,0526	0,0824
Mn	0,0052	0,0025	0,0026
Mg	0,8112	0,9261	0,9060
Ca	0,0000	0,0008	0,0007
Na	0,0000	0,0000	0,0000
K	0,0000	0,0000	0,0000
Cr	0,0001	0,0007	0,0000
Ni	0,0043	0,0005	0,0049
Fe/Mg	0,15	0,06	0,09

Table S2. Composition of cement in lithified till and moraine.

	Feragen													Leka				
Sample	Fer9-12	Fer9-12	Fer8-12	Fer8-12	Fer8-12	Fer8-12	Fer8-12	Fer8-12	Fer8-12	Fer8-12	Fer8-12	Fer7-12	Fer7-12	Fer7-12	LK14-G1a	LK14-G1a	LK14-G1a	LK14-G1a	LK14-G1b
SiO2	54,75	57,66	42,96	42,09	42,3	42,58	37,24	43,39	45,49	44,55	43,23	48,07	49,61	47,67	23,04	7,78	8,2	8,45	14,48
TiO2	0,05	0,01	0	0	0,01	0,01	0	0,01	0	0	0	0	0	0,01	0	0	0,01	0	0,01
Al2O3	3,16	0,75	0,18	0,15	0,77	0,36	0,03	1,27	0,01	0,13	0	0,39	1,55	0,06	12,89	18,47	18,68	18,42	16,42
FeO	0,43	0,12	0,15	0,07	0,1	0,13	0,16	0,6	0,06	0,16	0,05	0,29	0,38	0,88	0,73	0,14	0,15	0,36	0,17
MnO	0	0	0,05	0,01	0,02	0,02	0	0	0	0,01	0,03	0,09	0,09	0,07	0,05	0	0,04	0,02	0,06
MgO	27,38	26,76	38,95	37,45	37,98	38,81	35,59	37,75	38,37	36,63	39,77	32,19	32,12	35,11	24,59	36,78	34,42	34,24	32,52
CaO	0,28	0,06	0,07	0,07	0,02	0,01	0,05	0,06	0,1	0,06	0,05	0,23	0,11	0,41	0,73	0,1	0,11	0,04	0,14
Na2O	0,2	0	0	0,01	0	0	0,02	0,06	0,02	0,07	0,01	0,01	0,06	0,08	0,14	0	0,03	0	0,03
K2O	0,72	0,03	0,03	0,04	0,13	0,02	0,01	0,29	0,02	0,08	0,01	0,09	0,4	0,02	0,01	0	0	0,04	0,28
Cr2O3	0	0	0	0	0	0,04	0	0,02	0	0	0	0,05	0,14	0,02	0,04	0,07	0	0	0
NiO	0,01	0,01	0,07	0,05	0	0,06	0,02	0,03	0	0	0,03	0,08	0,01	0,05	0,22	0,15	0,18	0,42	0
Total	86,97	85,36	82,41	79,89	81,31	82,03	73,03	83,47	84,05	81,63	83,17	81,48	84,25	84,37	62,44	63,46	61,78	61,88	64,12
H2O	13,03	14,64	17,59	20,11	18,69	17,97	26,97	16,53	15,95	18,37	16,83	18,52	15,75	15,63	37,56	36,54	38,22	38,12	35,88

Sample	LK14-G1b	LK14-G1b	LK14-G1b	LK14-G1b	LK14-G1b	LK14-G1b	LK14-G1b	LK14-G1b	LK14-G1b	LK14-G1b	LK14-G1b	LK14-G1b	LK14-G1b	LK14-G1b	LK14-G1b	LK14-G1b	LK14-G1b	LK14-G1b
SiO2	44,33	40,95	45,08	43,4	42,94	38,24	41,13	26,22	27,47	11,37	17,45	42,79	13,01	35,13	41,83	33,58	48,01	45,05
TiO2	0,01	0	0	0	0,01	0	0	0,01	0,01	0,01	0,02	0	0	0	0,01	0	0,01	0,01
Al2O3	0,02	0,03	0,01	0,03	0,04	0,31	0,02	14,75	20,04	12,59	12,23	0,75	14,04	3,61	1,7	8,34	0,1	0,31
FeO	0,07	0,04	0,07	0,08	0,07	0,05	0,1	0,86	0,17	0,16	0,47	0,05	0,13	0,13	0,16	0,64	0,03	0,01
MnO	0	0	0	0	0,01	0,09	0,16	0,05	0,01	0,04	0,05	0	0,04	0,01	0	0,11	0	0,01
MgO	8,94	13,51	13,04	9,68	20,84	21,38	34,91	31,96	30,11	35,48	39,53	35,18	43,3	36,09	35,2	37,34	31,35	32,47
CaO	0,13	0,14	0,06	0,11	0,17	0,11	0,15	1,37	2,82	0	0,04	0,06	0,23	0,07	0,15	0,08	0,45	0,39
Na2O	0,04	0,02	0,01	0	0,01	0,01	0	0,75	1,96	0,05	0	0	0,35	0	0,03	0,01	0	0
K2O	0,05	0,01	0,06	0,04	0,02	0,05	0,02	0,13	0,02	0,09	0,02	0	0,02	0,03	0,44	0,02	0,02	0
Cr2O3	0,01	0,01	0	0	0	0	0,01	0	0,02	0	0,06	0	0,01	0	0,03	0,05	0	0,02
NiO	0,01	0,01	0	0,02	0	0,02	0,01	0,03	0,03	0,08	0,09	0,01	0,17	0,09	0	0,03	0,01	0
Total	53,63	54,67	58,32	53,31	64,09	60,19	76,67	76,1	82,65	59,85	69,95	78,75	71,31	75,03	79,58	80,2	79,97	78,25
H2O	46,37	45,33	41,68	46,69	35,91	39,81	23,33	23,9	17,35	40,15	30,05	21,25	28,69	24,97	20,42	19,8	20,03	21,75

Table S3. Whole rock composition of the blue(inner) and yellow(outer) weathering rinds and pristine peridotite(dark) and two samples of gravel.

Sample	F09.11			F12.11			R17.11		
	Dark	Blue	Yellow	Dark	Blue	Yellow	Dark	Blue	Yellow
SiO_2	36.31	39.62	38.86	35.33	37.82	39.54	34.86	37.29	38.5
Al_2O_3	0.14	0.19	0.22	0.06	0.11	0.14	0.48	0.32	0.47
Fe_2O_3							2.46	4.08	5.21
FeO							4.5	3.3	2.9
Fe_2O_3(T)	7.88	8.4	8.66	6.00	8.12	8.25	7.46	7.74	8.43
MnO	0.11	0.12	0.12	0.09	0.09	0.05	0.11	0.11	0.11
MgO	44.19	40.92	41.83	43.16	38.28	36.43	42.85	40.21	38.92
CaO	0.05	0.21	0.06	0.05	0.05	0.04	0.12	0.09	0.08
Na_2O	0.04	0.06	0.07	0.01	0.03	0.04	< 0.01	0.01	0.01
K_2O	0.02	0.02	0.04	< 0.01	< 0.01	0.02	< 0.01	< 0.01	< 0.01
TiO_2	0.001	0.002	0.002	0.001	0.001	0.002	0.004	0.002	0.003
P_2O_5	< 0.01	0.04	0.07	< 0.01	< 0.01	0.07	< 0.01	< 0.01	0.01
LOI	10.28	10.03	10.1	15.35	14.77	14.36	12.29	12.42	11.49
Total	99	99.61	100	100.1	99.3	98.94	99.2	98.59	98.37
CO_2							1.09	0.47	0.46
MgO/SiO_2	1.22	1.03	1.08	1.22	1.01	0.92	1.23	1.08	1.01
Mg0/Fe_2O_3T	5.61	4.87	4.83	7.19	4.71	4.42	6.38	5.76	5.13
MgO/NiO	153	113	131	114	90	72	167	145	141
Fe_2O_3/FeO							0.5	1.2	1.8
Sc	3	3	3	2	2	3	4	4	4
Be	< 1	< 1	< 1	< 1	< 1	< 1	<1	<1	<1
V	7	12	< 5	7	6	11	24	15	21
Cr	2090	2370	1930	520	700	480	7420	4840	6460
Co	114	121	123	103	109	93	100	106	103
Ni	2190	2760	2430	2870	3240	3840	1950	2100	2090
Cu	< 10	< 10	< 10	< 10	< 10	10	< 10	< 10	< 10
Zn	40	50	70	40	40	80	50	40	60
Ga	< 1	< 1	< 1	< 1	< 1	< 1	< 1	< 1	< 1
Ge	0.9	1	0.9	1	1.1	1.1	0.7	0.7	0.8
As	< 5	< 5	< 5	< 5	< 5	< 5	< 5	< 5	< 5
Rb	< 1	1	< 1	1	1	2	< 1	< 1	< 1
Sr	< 2	3	2	< 2	< 2	< 2	< 2	< 2	< 2
Y	< 0.5	< 0.5	< 0.5	< 0.5	1.1	0.6	< 0.5	< 0.5	< 0.5
Zr	2	2	< 1	3	3	3	< 1	< 1	< 1
Nb	< 0.2	< 0.2	0.3	< 0.2	< 0.2	< 0.2	< 0.2	< 0.2	< 0.2
Mo	< 2	< 2	< 2	< 2	< 2	< 2	< 2	< 2	< 2
Ag	< 0.5	< 0.5	< 0.5	< 0.5	< 0.5	< 0.5	< 0.5	< 0.5	< 0.5
In	< 0.1	< 0.1	< 0.1	< 0.1	< 0.1	< 0.1	< 0.1	< 0.1	< 0.1
Sn	< 1	< 1	< 1	< 1	< 1	< 1	< 1	< 1	< 1
Sb	0.9	1.2	1.1	0.9	1	0.8	< 0.2	< 0.2	< 0.2
Cs	< 0.1	< 0.1	< 0.1	< 0.1	0.1	0.1	< 0.1	< 0.1	< 0.1
Ba	2	7	13	4	4	10	11	< 3	< 3
La	0.05	0.22	0.17	0.11	0.24	1.19	1.26	0.3	0.07
Ce	0.07	0.21	0.29	0.08	0.16	0.87	2.46	0.44	0.16
Pr	< 0.01	0.04	0.04	0.01	0.05	0.14	0.27	0.05	0.02
Nd	< 0.05	0.12	0.15	< 0.05	0.19	0.55	0.92	0.13	0.06
Sm	< 0.01	0.05	0.02	0.05	0.11	0.12	0.1	0.03	0.02
Eu	0.011	0.013	0.011	0.005	0.005	0.015	0.023	0.005	0.005
Gd	< 0.01	0.02	0.04	< 0.01	0.18	0.04	0.04	0.01	< 0.01
Tb	< 0.01	< 0.01	< 0.01	< 0.01	0.03	0.01	< 0.01	< 0.01	< 0.01
Dy	< 0.01	0.03	0.02	< 0.01	0.22	0.1	0.02	< 0.01	< 0.01
Ho	< 0.01	< 0.01	< 0.01	< 0.01	0.04	0.02	< 0.01	< 0.01	< 0.01

Er	< 0.01	0.01	0.01	< 0.01	0.12	0.05	< 0.01	< 0.01	< 0.01
Tm	0.005	0.005	0.005	0.005	0.016	0.011	0.005	0.005	0.005
Yb	< 0.01	0.04	0.02	< 0.01	0.08	0.07	< 0.01	< 0.01	0.01
Lu	0.002	0.006	0.003	0.004	0.011	0.007	0.002	0.002	0.002
Hf	< 0.1	< 0.1	< 0.1	< 0.1	< 0.1	< 0.1	< 0.1	< 0.1	< 0.1
Ta	0.24	0.32	0.29	0.31	0.3	0.38	< 0.01	< 0.01	0.01
W	1.6	2.6	1.6	3.9	23.8	2.8	< 0.5	< 0.5	< 0.5
Tl	< 0.05	< 0.05	< 0.05	< 0.05	< 0.05	< 0.05	< 0.05	< 0.05	< 0.05
Pb	< 5	< 5	6	< 5	< 5	7	< 5	< 5	< 5
Bi	< 0.1	< 0.1	< 0.1	< 0.1	< 0.1	< 0.1	< 0.1	< 0.1	< 0.1
Th	< 0.05	< 0.05	< 0.05	< 0.05	< 0.05	0.11	0.17	< 0.05	< 0.05
U	< 0.01	0.02	0.02	0.02	0.03	0.07	< 0.01	< 0.01	< 0.01

Table S3. Continued

Sample	R3-11			FW2.14		FW12.14			R17.11a	R7.11b
	Dark	Blue	Yellow	Dark	Yellow	Dark	Blue	Yellow	Gravel	Gravel
SiO_2	37.68	38.61	40.34	35.19	37.08	33.57	35.93	35.91	40.00	41.31
Al_2O_3	0.09	0.09	0.07	0.20	0.26	0.13	0.16	0.24	0.56	0.81
Fe_2O_3	0.72	0.46	3.1						4.73	5.21
FeO	5.4	5.7	4.7						2.60	2.50
Fe_2O_3(T)	6.71	6.79	8.31	7.75	8.38	7.69	8.46	8.58	7.62	7.99
MnO	0.11	0.11	0.11	0.10	0.11	0.10	0.11	0.11	0.12	0.12
MgO	46.4	45.18	45.2	44.43	42.97	45.84	44.46	43.77	38.71	35.08
CaO	0.10	0.07	0.07	0.12	0.07	< 0.01	< 0.01	< 0.01	0.07	0.11
Na_2O	< 0.01	0.01	< 0.01	0.04	0.06	0.06	0.15	0.09	< 0.01	0.04
K_2O	< 0.01	< 0.01	< 0.01	0.02	0.03	0.02	0.03	0.05	< 0.01	0.14
TiO_2	0.003	0.002	0.002	0.00	0.00	0.00	0.00	0.00	0.01	0.02
P_2O_5	< 0.01	< 0.01	< 0.01	< 0.01	< 0.01	< 0.01	< 0.01	< 0.01	< 0.01	0.02
LOI	6.27	6.04	5.49	10.86	10.37	10.73	10.29	9.49	12.53	13.16
Total	98	97.55	100.2	98.70	99.32	98.13	99.58	98.23	99.90	99.06
CO_2	0.01	0.25	0.25						0.15	0.88
MgO/SiO_2	1.23	1.17	1.12	1.26	1.16	1.37	1.24	1.22	0.97	0.85
$Mg0/Fe_2O_3T$	7.67	7.39	6.03	5.73	5.13	5.96	5.26	5.10	5.08	4.39
MgO/NiO	196	197	119	145	126	138	129	123	133	134
Fe_2O_3/FeO	0.1	0.1	0.7						1.8	2.1
Sc	3	3	3	5	4	3	3	3	7	6
Be	<1	<1	<1	< 1	< 1	< 1	< 1	< 1	<1	<1
V	10	10	7	13	13	6	6	< 5	25	23
Cr	2650	2920	2660	2960	2980	3090	2530	2480	5000	3590
Co	87	86	124	113	121	118	121	127	110	102
Ni	1800	1740	2880	2330	2590	2520	2610	2700	2210	1980
Cu	< 10	< 10	< 10	< 10	< 10	< 10	< 10	< 10	< 10	< 10
Zn	< 30	< 30	40	40	50	40	60	50	70	60
Ga	< 1	< 1	< 1	< 1	< 1	< 1	< 1	< 1	< 1	< 1
Ge	0.7	0.6	0.9	< 0.5	< 0.5	< 0.5	< 0.5	< 0.5	1.1	0.9
As	< 5	< 5	< 5	< 5	< 5	< 5	< 5	< 5	< 5	< 5
Rb	< 1	< 1	< 1	< 1	< 1	< 1	< 1	< 1	< 1	4
Sr	< 2	< 2	< 2	< 2	< 2	< 2	< 2	2	< 2	< 2
Y	< 0.5	< 0.5	< 0.5	< 0.5	< 0.5	< 0.5	< 0.5	< 0.5	< 0.5	< 0.5
Zr	< 1	< 1	< 1	1	1	< 1	2	2	< 1	9
Nb	< 0.2	< 0.2	< 0.2	< 0.2	< 0.2	< 0.2	< 0.2	< 0.2	< 0.2	< 0.2
Mo	< 2	< 2	< 2	< 2	< 2	< 2	< 2	< 2	< 2	< 2
Ag	< 0.5	< 0.5	< 0.5	< 0.5	< 0.5	< 0.5	< 0.5	< 0.5	< 0.5	< 0.5

In	< 0.1	< 0.1	< 0.1	< 0.1	< 0.1	< 0.1	< 0.1	< 0.1	< 0.1	< 0.1
Sn	< 1	< 1	< 1	< 1	< 1	< 1	< 1	< 1	< 1	< 1
Sb	< 0.2	< 0.2	< 0.2	< 0.2	< 0.2	< 0.2	< 0.2	< 0.2	< 0.2	0.2
Cs	< 0.1	< 0.1	< 0.1	< 0.1	< 0.1	< 0.1	< 0.1	< 0.1	< 0.1	0.1
Ba	4	< 3	< 3	4	4	3	3	4	3	27
La	0.62	0.18	0.15	< 0.05	0.06	< 0.05	0.42	0.1	0.23	0.49
Ce	1.13	0.32	0.11	< 0.05	0.09	0.07	0.59	0.2	0.36	1.07
Pr	0.12	0.04	0.01	< 0.01	0.01	< 0.01	0.03	0.02	0.03	0.12
Nd	0.38	0.16	0.05	< 0.05	0.07	0.07	0.2	0.08	0.11	0.46
Sm	0.05	0.02	< 0.01	< 0.01	< 0.01	< 0.01	0.02	0.01	0.02	0.08
Eu	0.014	<0.005	0.005	0.005	0.005	0.005	0.005	0.005	0.005	0.019
Gd	0.04	0.01	< 0.01	< 0.01	< 0.01	< 0.01	0.03	< 0.01	0.01	0.08
Tb	< 0.01	< 0.01	< 0.01	< 0.01	< 0.01	< 0.01	< 0.01	< 0.01	< 0.01	0.01
Dy	0.02	0.01	0.01	< 0.01	0.02	0.02	0.02	0.02	< 0.01	0.09
Ho	< 0.01	< 0.01	< 0.01	< 0.01	< 0.01	< 0.01	< 0.01	< 0.01	< 0.01	0.02
Er	0.01	< 0.01	< 0.01	< 0.01	< 0.01	< 0.01	< 0.01	0.01	< 0.01	0.05
Tm	0.005	0.005	0.005	0.005	0.005	0.005	0.005	0.005	0.005	0.008
Yb	0.02	< 0.01	0.01	0.03	0.01	0.02	0.03	0.03	0.01	0.05
Lu	0.004	0.002	0.003	0.002	0.006	0.002	0.004	0.002	0.003	0.01
Hf	< 0.1	< 0.1	< 0.1	< 0.1	< 0.1	< 0.1	< 0.1	< 0.1	< 0.1	0.2
Ta	0.01	< 0.01	< 0.01	< 0.01	< 0.01	< 0.01	< 0.01	< 0.01	< 0.01	0.02
W	< 0.5	< 0.5	< 0.5	0.5	< 0.5	< 0.5	< 0.5	< 0.5	< 0.5	< 0.5
Tl	< 0.05	< 0.05	< 0.05	< 0.05	< 0.05	< 0.05	< 0.05	< 0.05	< 0.05	< 0.05
Pb	< 5	< 5	< 5	< 5	< 5	< 5	< 5	< 5	< 5	< 5
Bi	< 0.1	< 0.1	< 0.1	< 0.1	< 0.1	< 0.1	< 0.1	< 0.1	< 0.1	< 0.1
Th	0.08	0.05	0.05	< 0.05	< 0.05	< 0.05	< 0.05	< 0.05	< 0.05	0.15
U	0.01	0.04	0.02	< 0.01	< 0.01	< 0.01	< 0.01	0.01	< 0.01	0.04

Table S4. Composition of till from trench through mudboil(FER18/15 and 19/15) and nearby tillite (FER21/15).

	FER18/15<br>Trench	FER19/15<br>Trench	FER21/15<br>Tillite
SiO2	87,72	89,58	72,81
Al2O3	4,48	4,23	4,25
Fe2O3(T)	1,54	1,42	1,01
MnO	0,02	0,02	0,01
MgO	0,79	0,63	9,98
CaO	0,26	0,3	0,24
Na2O	0,69	0,7	0,56
K2O	2,11	1,99	1,9
TiO2	0,2	0,21	0,16
P2O5	0,04	0,03	0,03
LOI	0,7	0,52	9,4
Total	98,55	99,61	100,4
Be	< 1	< 1	< 1
V	15	13	12
Cr	110	230	30
Co	4	4	3
Ni	40	40	60
Cu	< 10	< 10	< 10
Zn	< 30	< 30	< 30
Ga	5	4	4

Ge	1	0,8	1,1
As	< 5	< 5	< 5
Rb	53	49	51
Sr	39	45	36
Y	8,7	9,1	9,5
Zr	164	188	136
Nb	4	3,8	4,1
Mo	< 2	4	< 2
Ag	0,7	0,8	0,5
In	< 0.1	< 0.1	< 0.1
Sn	< 1	< 1	< 1
Sb	0,4	0,4	0,5
Cs	0,8	0,7	0,8
Ba	387	376	344
La	11,3	10,1	9,88
Ce	22,6	20,5	19,1
Pr	2,58	2,41	2,3
Nd	9,78	9,01	8,97
Sm	1,98	1,85	1,64
Eu	0,421	0,439	0,432
Gd	1,64	1,56	1,51
Tb	0,27	0,27	0,26
Dy	1,66	1,62	1,54
Ho	0,32	0,33	0,33
Er	0,95	0,97	0,93
Tm	0,147	0,151	0,147
Yb	0,97	1,02	1,02
Lu	0,151	0,159	0,156
Hf	3,5	4,1	3,2
Ta	0,55	0,52	0,53
W	1,5	1,4	1,1
Tl	0,23	0,23	0,22
Pb	7	7	7
Bi	< 0.1	< 0.1	< 0.1
Th	2,66	2,23	1,99
U	0,83	0,85	0,73
